# Toxic Effects of Chronic Exposure to High-Fat Diet and Arsenic on the Reproductive System of the Male Mouse 

**Published:** 2019-12

**Authors:** Akram Ahangarpour, Ali Akbar Oroojan, Soheila Alboghobeish, Layasadat Khorsandi, Mitra Moradi

**Affiliations:** 1 Diabetes Research Center, Health Research Institute, Department of Physiology, Faculty of Medicine, Ahvaz Jundishapur University of Medical Sciences, Ahvaz, Iran; 2 Department of Physiology, Faculty of Medicine, Student Research Committee, Ahvaz Jundishapur University of Medical Sciences, Ahvaz, Iran; 3 Department of Physiology, Faculty of Medicine, Dezful University of Medical Sciences, Dezful, Iran; 4 Department of Pharmacology, Faculty of Pharmacy, Student Research Committee, Ahvaz Jundishapur University of Medical Sciences, Ahvaz, Iran; 5 Department of Anatomical Sciences, Faculty of Medicine, Cellular and Molecular Research Center, Ahvaz Jundishapur University of Medical Sciences, Ahvaz, Iran; 6 Diabetes Research Center, Health Research Institute, Ahvaz Jundishapur University of Medical Sciences, Ahvaz, Iran

**Keywords:** Arsenic, High Fat Diet, Gonadotropin, Sperm, Testis

## Abstract

**Objective:** Obesity is associated with reproductive disorders. Arsenic disrupts male reproduction by direct effects on the male gonads or androgens secretion. So, the present study was conducted to evaluate the toxic effects of chronic concomitant administration of high-fat diet (HF) and arsenic on the reproductive system of the male mouse.

**Materials and methods:** In this experimental study, 72 adult male mice were randomly divided into 6 groups: low-fat diet (LF0), LF+arsenic 25 ppm, LF+arsenic 50ppm, HF0, HF+arsenic 25 ppm and, HF+arsenic 50 ppm. 24 hours after the last experimental day, plasma samples, the cauda of epididymis and testis were prepared and removed for hormonal, sperm count and histopathological assessments.

**Results:** Testis weight and volume increased in HF0 than other groups except for LF0. Plasma LH and testosterone levels decreased in LF50, HF0, HF25, and HF50 compared to LF0. A similar effect was observed in plasma FSH levels of HF0, HF25 and HF50 groups compared with LF0. Plasma level of estradiol increased in LF50 versus to other groups. Testosterone to estradiol ratio and sperm count decreased in all groups compared to LF0. Reduced interstitial cells and large numbers of vacuoles were observed in germinal epithelium of HF0 group, that these changes were more intense in both concentrations of arsenic-treated mice.

**Conclusion:** Present study indicated that chronic exposure to HF and arsenic-induced hypogonadotropic hypogonadism concomitant with sperm count reduction and testicular damage.

## Introduction

The infertility prevalence is approximately 15 %, that 30 to 50 % of this rate belongs to male factors (1). The World Health Organization (WHO) estimates, more than 500 million adults are obese and approximately 3 million people die as a result of obesity per year. Obesity is associated with several complications such as insulin resistance, type 2 diabetes mellitus, hypertension, dyslipidemia, and reproductive disorders (2). Obese and overweight men exhibit a high prevalence of infertility associated with metabolic and reproductive hormonal disorders (3). These events were characterized by lowered total and free testosterone, gonadotropins, and enhanced plasma estrogen levels, which have a positive relationship with the degree of obesity. High level of estradiol has occurred as a result of increased peripheral aromatization of androgens in these cases. Further, a decrease in testosterone to estrogen ratio has occurred through male infertility. Previous research showed that testosterone to estrogen ratio is significantly lower in infertile men than fertile control subjects (4). Long-term treatment with high-fat diet provoked obesity that leads to an altered function of the pituitary-testicular axis and developed hypogonadotropic hypogonadism (5). The same alteration has occurred in the availability of gonadotropin receptors, such as luteinizing hormone (LH) and follicle stimulating hormone (FSH) which, lead to reduce spermatogenesis in seminiferous tubules and testosterone production from the Leydig cells (2). It has been reported that high plasma levels of cholesterol or triglycerides in the high-fat diet (fat in 58% of total daily calorie) are associated with testicular dysfunction which may lead to male infertility. Also, Diet-induced hyperlipidemia in rabbits has been shown adverse effects on sperm concentration (6). Arsenic (As) is a nonessential trace element has drawn increasing attention by scientists as a major contaminant of drinking water and, human exposure to this metal through the drinking water is rising due to pollution from industrial operations since 1978 (7). A large number of people are encountered with arsenic consumption and toxicity via drinking of contaminated water in numerous regions of the world such as China, India, some Central and South American countries and West Bengal (8). The origin of most of the arsenic pollution was reported in Kurdistan, East Azarbaijan, and Kerman provinces in Iran and the highest contaminated area is the Kerman province with the mean concentration of 545 (μg/l), that is more than WHO and National Standard limits (NSI). Arsenic (As) disrupts male reproduction by direct effects on the male gonads or modulates pituitary activity, which causes to disruption of androgens secretion from Leydig cells, decrease testosterone levels within the testes and spermatogenesis in seminiferous tubules. It has been reported that arsenic-induced oxidative stress that leads to testicular cell death or apoptosis and low sperm count or concentration (9, 10). Also, arsenic administration can induce male infertility via several mechanisms including reduction of spermatogenesis, destruct the testosterone pathway, increase oxidative stress, inflammation, and activation of heat shock proteins in testes (11). The previous study indicated 12-weeks exposure to arsenite and arsenate through drinking water produced different patterns of dyslipidemia identified by inhibition of reverse cholesterol transport and the increase free fatty acid (FFA) in plasma. Hence, these two denominators induced lipotoxic and non-lipotoxic dyslipidemia that cause cardiovascular disease (12). Our previous study revealed that combined exposure of HFD and As aggravate diabetes complications due to the induced liver oxidative stress and damage, failure of the pancreatic β cells function, hepatotoxicity and hypothyroidism through the reduction of thyroid hormones and enhancement of plasma TSH and T3 uptake (13, 14). 

It was revealed that small animals such as mouse might be less susceptible than human to arsenic toxicity due to a faster metabolism and clearance of this agent. Moreover, a recent study indicated that 10 times higher concentration of drinking water arsenic is required (50 ppm) to achieve liver arsenic concentrations similar to those seen in human exposure to arsenic in West Bengal (15). Therefore, according to the high prevalence of obesity, the presence of arsenic in drinking water in society or Iran and, the influence of these factors on the male reproductive system, the present study was conducted to evaluate the effects of chronic high-fat diet in combination with arsenic consumption on the reproductive system of the male mouse.

## Materials and methods

***Preparation of animal: ***In this experimental study, 72 male Naval Medical Research Institute (NMRI) mice (30-35 g) were selected according to the previous research (16) and obtained from the animal facility of Ahvaz Jundishapur University of Medical Science (AJUMS), which is fully accredited by AJUMS animal care guidelines with an ethics committee grantee No. (IR.AJUMS.REC.1395.405). After one-week acclimatization, the animals were kept at polycarbonate cages with corncob bedding in 20 ± 4 °C temperature with a 12h light / 12h dark cycle and 10% humidity. A low-fat diet (LFD; 11% fat, 16% protein, and 73% carbohydrate kcal/g) or high-fat diet (HFD; 58% fat, 16.5% protein, and 25.5% carbohydrate kcal/g) were purchased from Javaneh Khorasan lab. Iran. (14) According to the grain-based diet contained 19.5-28.6 ppb arsenic (mainly iAs) and its possibly compromising the study design. To avoid this problem a purified diet without grain components has been used. The level of arsenic in the high-fat diet was 10 ppb and in the low-fat diet was 15 ppb which consist of a very lower concentration of arsenic in comparison with examined concentrations (25 and 50 ppm). Therefore, mice drank diH2O or diH2O plus arsenite (Sigma Chemical Co, USA) in doses of 25 or 50 ppm in the present study. Water containing arsenite was replaced every 3 days to minimize its oxidation. So, mice were divided into 6 groups (n=12 in each group): control (LF0; received low fat diet for 20 weeks), LF25 (received low fat diet concomitant with arsenic 25 ppm for 20 weeks), LF50 (received low fat diet concomitant with arsenic 50 ppm for 20 weeks), HF0 (received high fat diet for 20 weeks), HF25 (received high fat diet concomitant with arsenic 25 ppm for 20 weeks) and, HF50 (received high fat diet concomitant with arsenic 50 ppm for 20 weeks) (15).

***Experimental assessment: ***At the end of experiment, the animals were anesthetized by ether and plasma samples were prepared by cardiac puncture blood collection and centrifuging at 3500 rpm for 20 min. All samples were kept at −80 °C until the hormonal measurements were performed. Then, the right testes of the mice were removed instantly and testicular morphology was assessed in each group (17). Volume of testis was analyzed by using the following formula: volume = (D^2 ^/ 4 × π) L × K (length (L), width (D), K = 0.9, π = 3.14) (18).

***Sperm assessment: ***The cauda of epididymis was separated and minced it to the small pieces in Petri dish containing 6 mL 0.9% normal saline. A drop of Petri dish solution was transferred into Neubauer hemocytometer lam (HBG, Company, Germany) (Tiefe depth profondeur 0.100 mm and 0.0025 mm2 area) for sperm counting. Then this variable has been counted manually in the white blood cell chambers under light microscopy (Olympus light microscope Tokyo, Japan). Finally, according to the 6 mL of 0.9% normal saline administration for sperm sample dilution, it has been used as a dilution coefficient and, the data of sperm counting were expressed as the millions of sperm per milliliter (17). 

***Hormonal assessment: ***Plasma luteinizing hormone (LH), follicle-stimulating hormone (FSH), and testosterone levels have been measured by enzyme-linked immunosorbent assay (ELISA) method with commercial assay kits (DRG Instruments GmbH, Germany) and, hormone detection sensitivity per assay tube of each kit was 0.856 mIU/mL for LH, 1.27 mIU/mL for FSH, and 0.287 nmol/L for testosterone.

***Histopathological assessment: ***Six microscopy slides per animal were examined for interstitial cells number and signs of germ cell degeneration including the following histopathological alterations: detachment, sloughing, and vacuolization. For each treatment, the average percentage of normal and regressed tubules was determined. Average percentages were calculated for each sample by dividing the number of round tubules with a histopathology index (vacuolization, detachment, sloughing) or normal tubules in a randomly microscopic field by the total number of round tubules in the same field and the result multiplied by 100. For each slide, the mean of three fields was considered (19, 20). 

***Morphometry: ***Motic Images Plus 2.0 image analysis software has been used for diameters of the seminiferous tubules and the lumen diameter measurement. The height of the seminiferous epithelium was calculated by subtracting the lumen diameter from the tubule diameter. 150 tubules were analyzed for each animal. Ultimately, slides reading has been used by a “blind” method (21).

***Statistical Assessment: ***Data were statistically analyzed using the Statistical Package for the Social Sciences (SPSS) software and the comparison between groups was assessed using one-way analysis of variance (ANOVA) followed by post hoc least significant difference (LSD) tests. Values are presented as mean ± standard error of the mean (SEM) and p < 0.05 were considered statistically significant.

## Results

***Body weight and testicular morphology of the mice: ***As shown in Table 1, body weight increased in HF0 compared with LF0, but this variable decreased in HF25 and HF50 versus to HF0. However, testis weight decreased in LF25, HF25, HF50 and LF50 compared to the LF0 group, but this variable increased in HF0 in comparison with other groups except for LF0. Testis length assessment indicated a significant decrease in LF25, HF25, HF50 and LF50 when compared to LF0. Further, this testicular variable increased in HF0 in comparison with other groups except for LF0. The width of testis decreased in HF50 versus to LF0 group and administration of high-fat diet increased in HF0 compared to LF25, LF50 and HF50. Testis volume revealed a significant decrease in LF25, LF50 and HF50 in comparison with LF0 group. Ultimately, high fat diet consumption increased the volume of the testis in HF0 compared to LF25, LF50, and HF50. 

**Figure 1:**
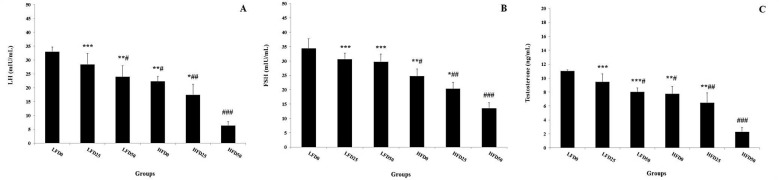
Effect of high fat diet and arsenic exposure on plasma LH, FSH and Testosterone levels. Data are presented as Mean ± SEM; n = 12. # P<0.05, ## P<0.01, and ### P<0.001 compared to the LF0. * P<0.05, ** P<0.01, and *** P<0.001 compared to the HF50. LF: Low fat diet; HF; High fat diet.

**Figure 2:**
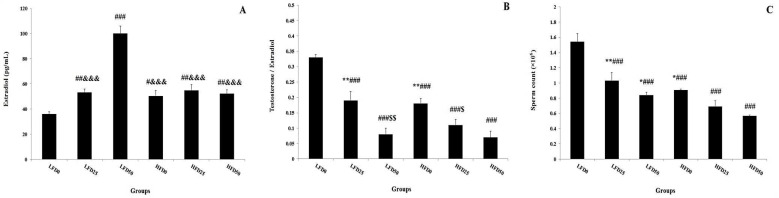
Effect of high fat diet and arsenic exposure on plasma estradiol, testosterone to estradiol ratio and sperm count. Data are presented as Mean ± SEM; n = 12.

***High fat diet and arsenic exposure and reproductive hormones: ***The results of gonadotropins measurement showed a significant decrease in plasma LH levels in LF50, HF0, HF25 and HF50 compared to LF0. Also, this hormone significantly decreased in HF50 when compared with LF25, LF50, HF0 and HF25 groups (Figure 1A). Also, the same effects were observed in plasma FSH levels of HF0, HF25 and HF50 groups in comparison with LF0. The plasma levels of this hormone decreased in HF50 versus to LF25, LF50, HF0 and HF25 groups (Figure 1B). Testosterone assessment showed a significant decrease in LF50, HF0, HF25 and HF50 compared to LF0 group. Concomitant administration of high-fat diet and arsenic 50ppm indicated a remarkable decrease in plasma levels of testosterone when compared to LF25, LF50, HF0 and HF25 groups (Figure 1C). Plasma level of estradiol revealed an increase in LF50 versus to other groups and the same effect was observed in HF0, LF25, HF25 and HF50 compared to LF0 (Figure 2A). Testosterone to estradiol ratio showed a significant decrease in all groups when compared to LF0 and, high fat diet in combination with arsenic 50ppm exposure reduced this ratio compared to LF25 and HF0. Ultimately, a similar effect was observed in LF50 and HF25 in comparison with HF0 group (Figure 2B). 

Sperm count decreased in all groups compared to LF0 and, administration of high fat diet concomitant with arsenic 50ppm revealed more reduction in this variable when compared to HF0 and LF50 (Figure 2C).

**Figure 3:**
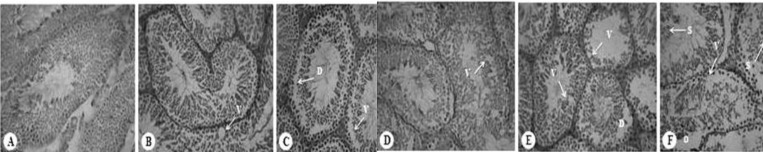
Effect of high fat diet and arsenic exposure on testicular histopathology. A: LF0, B: LF25, C: LF50, D: HF0, E: HF25, F: HF50. (H & E; Magnification: ×40). LF: Low fat diet; HF: High fat diet; D: Detached; S: Slough; V: Vacuole.

***Assessment of sperm count and testicular histopathology: ***Testicular sections from the LF0 group showed normal spermatogenesis with a low incidence of detached, sloughed or vacuolized seminiferous tubules. Normal architecture of the seminiferous tubules and intact germinal epithelium were also observed in LF25 group. In LF 50 numerous vacuoles and detachment were observed in germinal epithelium. Large numbers vacuoles were observed in germinal epithelium of HF0 group. In the HF25 group, varying degrees of germ cell degenerative changes occurred, ranging from loss of elongated spermatids, disorganization of germ cell layers, detachment and sloughing to vacuolization of the seminiferous tubules, contributing to eventual atrophy and, these changes were more intense in HF50 treated mice (Figure 3). As shown in Table 2, reduced numbers of interstitial cells were observed in LF25, LF50, HF0, HF25 and HF50 compared to LF0 group. A similar effect has occurred in HF25 and HF50 when compared to HF0 group. Also, decreased the normal appearance of seminiferous tubules and increased detachment of them were observed in LF50, HF0, HF25 and HF50 compared to LF0 group. Moreover, the same effect was observed in HF25 and HF50 groups when compared to HF0. The sloughed seminiferous tubules of LF50, HF0, HF25 and HF50 groups significantly increased versus to LF0. This variable assessment revealed the same changes in HF25 and HF50 when compared to HF0 group. The vacuolization of these tubules increased in LF50, HF0, HF25 and HF50 in comparison with LF0. Also, the same effect has occurred in HF25 and HF50 compared to HF0 group. Seminiferous tubule diameter and epithelium height were decreased in LF50, HF0, HF25 and HF50 compared to LF0 group. Further, the same effect was observed in HF25 and HF50 when compared to HF0 group.

## Discussion

The present data indicate that arsenic reduced testicular weight, volume and seminiferous tubule epithelium height and diameter in a dose-dependent manner and this agent have more effect on body weight and testicular morphology at the high dose in high-fat diet fed mice. As high-fat diet consumption didn’t change testicular weight and volume it could be suggested that arsenic administration is more potent than this diet on testicular properties. However, acute administration of arsenic couldn’t change the testicular weight  (22) , but this variable reduced after 5-week arsenic utilization in male mice (23). So, the duration of arsenic exposure is an important reason to produce a testicular weight reduction. Hence, the present study suggested that chronic administration of arsenic plays an important role in testicular weight and volume reductions. 

**Table 1:** Effect of high fat diet and arsenic exposure on testicular morphology

**Groups**	**Testicular factors**									
	**Body weight ** **(g)**	**p-value**	**Testis weight ** **(mg)**	**p-value**	**Testis length ** **(mm)**	**p-value**	**Testis width ** **(mm)**	**p-value**	**Testis volume ** **(mm** ^3^ **)**	**p-value**
LF0	37.65 ± 2.9	-	125.71 ± 3.04	-	8.50 ± 0.18	-	4.37 ± 0.18	-	116.74 ± 10.93	-
LF25	35.28 ± 4.7	= 0.634, < 0.001	103.10 ± 3.79	< 0.001, < 0.001	7.50 ± 0.32	= 0.009, < 0.001	4.00 ± 0.26	= 0.284, = 0.036	87.25 ± 11.35	= 0.048, =0.013
LF50	34.63 ± 3.1	= 0.207, < 0.001	109.00 ± 2.51	= 0.002, < 0.001	7.66 ± 0.21	= 0.040, = 0.002	3.85 ± 0.14	= 0.156, = 0.017	80.07 ± 6.26	= 0.027, = 0.007
HF0	47.21 ± 8.3	< 0.001	134.83 ± 4.48	= 0.150	9.00 ± 0.26	= 0.177	4.75 ± 0.36	= 0.284	127.17 ± 13.81	= 0.500
HF25	35.58 ± 3.2	= 0.189, < 0.001	99.57 ± 6.43	< 0.001, < 0.001	7.50 ± 0.18	= 0.009, < 0.001	4.75 ± 0.25	= 0.284, = 0.156	112.13 ± 11.90	= 0.764, = 0.347
HF50	30.10 ± 2.2	< 0.001, < 0.001	95.28 ± 2.33	< 0.001, < 0.001	7.37 ± 0.32	= 0.004, < 0.001	3.62 ± 0.18	= 0.036, = 0.002	71.00 ± 8.59	= 0.004, = 0.001

Data are presented as Mean ± SEM; n = 12. The first p-value represents compared to the LF0 and the second p-value represents compared to the HF0. LF: Low fat diet; HF; High fat diet.

**Table 2:** Effect of high fat diet and arsenic exposure on leydig cells number and seminiferous tubules changes.

**Groups**	**Percentage of tubules**								**Interstitial cells**		**STD**		**SHE**	
	**Normal**	**p-value**	**Detached**	**p-value**	**Sloughed**	**p-value**	**Vacuolized**	**p-value**	**Number (10** ^6^ **)**	**p-value**	**(10** ^-6^ ** m)**	**p-value**	**(10** ^-6^ ** m)**	**p-value**
LF0	96.3 ± 4.2	-	1.1 ± 0.3	-	0.1 ± 0.03	-	1.8 ± 0.4	-	32.3 ± 3.3	-	212 ± 14.2	-	69.1 ± 4.3	-
LF25	93.3 ± 7.8	= 0.416, = 0.271	2.5 ± 1.5	= 0.286, = 0.374	2.8 ± 1.2	= 0.068, = 0.089	3.6 ± 1.3	= 0.571, = 0.094	24.4 ± 2.5	= 0.038, 0.442	201 ± 7.8	= 0.376, = 0.089	63.7 ± 4.5	= 0.312, 0.091
LF50	80.3 ± 5.8	= 0.039, = 0.325	9.5 ± 1.8	= 0.014, = 0.022	3.8 ± 0.2	= 0.029, = 0.036	12.6 ± 1.6	= 0.005, 0.387	21.1 ± 1.8	= 0.033, 0.352	180.3 ± 11.8	= 0.047, = 0.249	56.5 ± 5.8	= 0.040, = 0.145
HF0	87.1 ± 5.2	= 0.046	2.1 ± 0.3	= 0.042,	0.3 ± 0.03	= 0.047	10.8 ± 0.4	= 0.006	23.7 ± 2.1	= 0.027	177.1 ± 10.2	= 0.041	54.1 ± 3.3	=0.036
HF25	68.8 ± 5.2	=0.034, =0.041	16.5 ± 1.5	= 0.003, = 0.004	4.8 ± 1.2	= 0.001, = 0.003	21.6 ± 1.3	< 0.001, = 0.008	18.1 ± 1.4	= 0.008, = 0.038	168.8 ± 9.2	= 0.017, = 0.022	46.5 ± 2.5	= 0.028, = 0.037
HF50	38.8 ± 3.7	=0.005, =0.007	24.5 ± 3.5	< 0.001, < 0.001	8.8 ± 2.1	< 0.001, < 0.001	37.4 ± 5.3	< 0.001, < 0.001	11.2 ± 1.6	< 0.001, = 0.008	109.8 ± 8.7	= 0.006, = 0.009	37.5 ± 3.5	= 0.005, = 0.008

Data are presented as Mean ± SEM of percentage of tubules; n = 12. The first p-value represents compared to the LF0 and the second p-value represents compared to the HF0. LF: Low fat diet; HF; High fat diet. STD: seminiferous tubule diameter; SEH: seminiferous epithelium height.

Hypergonadotropic hypogonadism, as primary hypogonadism, has been occurred by the primitive gonadal disease and characterized by low/normal plasma levels of testosterone and high FSH and LH levels. Hypogonadotropic hypogonadism, as secondary hypogonadism, is characterized by decreased plasma testosterone levels associated with normal or low plasma levels of FSH and LH. Recently, it was revealed that an impairment of gonadotropin secretion and, a reduced efficiency of spermatogenesis produced a different condition of the classical causes of secondary hypogonadism which named as functional hypogonadotropic hypogonadism (24). Our results of concomitant administration of high-fat diet and arsenic 50 ppm revealed a remarkable reduction in gonadotropins and testosterone levels and, demonstrated that hypogonadotropic hypogonadism has been occurred. Previous in vivo and in vitro research showed that arsenic could increase estrogen-like chemical and estrogen level (25, 26). Arsenic 50 ppm increased plasma estradiol level in low-fat diet consumed mice. Testosterone to estradiol ratio reduced in HFD fed mice and administration of arsenic intense this effect in both LFD and HFD consumed mice in a dose-dependent manner. Hence, it was revealed that arsenic and HFD concomitant administration is more potent on testosterone than estradiol. Sperm count assessment showed an impressive decrease in HFD fed mice and administration of arsenic 50ppm induced more reduction in this variable. Further, alone arsenic could diminish in sperm numbers dose-dependently.

High-fat feeding disrupted LH and testosterone daily rhythms concomitant with lowered plasma level of testosterone. Hence, it has been demonstrated that obesity plays an important role in the hypophysial–gonadal axis disorder (27). Consist with the present results Ibrahim et al, demonstrated a significant reduction in serum LH, FSH, testosterone levels, epididymal sperm count and elevation of estradiol levels in high-fat diet consumed male rat (28). One of the main mechanisms of these events is of testosterone to estradiol consequence conversion via the rise of aromatase expression at high levels in the adipose tissue. Moreover, estrogen has receptors in hypothalamic nuclei and pituitary gonadotrope, hence it’s assumed that this hormone can effect on the gonadotropin-releasing hormone (GnRH) pulses, induce suppression of both LH and FSH secretion through the influence on hypothalamic-pituitary axis and produce hypogonadotropic hypogonadism (29, 30). According to another suggestion, estradiol alters the spermatogenesis due to a direct effect on the testicular environment (31). FSH is an essential hormone to stimulate synthesize of androgen binding protein and spermatozoa production from Sertoli cells. Hence, the decreased level of FSH may interfere in epididymal sperm count reduction in addition to hypogonadotropic hypogonadism (32). 

Arsenic-induced gonadal dysfunction through the declined plasma concentrations of LH, FSH, and testosterone. This agent might exert a direct testicular inhibitory action by a decrease of Leydig cells, or effect on the hypothalamic-pituitary axis results in the reduction of plasma LH and FSH concentrations. LH is an essential hormone for normal function of Leydig cells. Hence, plasma reduction of this hormone can impair these cells function and result in a consequent reduction of testosterone production as a prerequisite hormone for normal spermatogenesis. Ultimately, these events lead to a decrease in spermatogenesis and epididymal sperm count. It was revealed that exposed to drinking-water arsenic for 5 weeks could decrease plasma levels of testosterone and gonadotropins, sperm counts and testicular weight in male mice (33). Therefore, the results of present study revealed that 20-week administration of arsenic destroyed male reproductive system function or construction similar to previous studies and, chronic coadministration of high-fat diet and arsenic produced more sperm number reduction and hypogonadotropic hypogonadism compared to alone utilization of them. The formation of large vacuoles at the locations of spermatogonia and primary spermatocytes may be formed by the fluid within the ground cytoplasm of the germinal epithelial cells and could be attributed to the degeneration and detachment of the spermatogenic cells and Sertoli cell injury (34, 35). Also, consists with previous studies it was revealed that chronic coadministration of HFD and arsenic-induced germ cell degeneration, disorganization of germ cell layers, detachment and sloughing to vacuolization of the seminiferous tubules, which are associated with hypogonadism and decreased spermatogenesis.

## Conclusion

In conclusion, the present study indicated that chronic exposure to arsenic in combination with high fat diet-induced reproduction toxic effects through the functional hypogonadotropic hypogonadism that results to gonadotropin and testosterone decreases concomitantly with sperm count reduction and testicular alteration such as germ cell degenerative, detachment and sloughing to vacuolization of the seminiferous tubules.
